# Probing the Timing Recruitment of Broca’s Area in Speech Production for Mandarin Chinese: A TMS Study

**DOI:** 10.3389/fnhum.2018.00133

**Published:** 2018-04-10

**Authors:** Qian Zhang, Banglei Yu, Junjun Zhang, Zhenlan Jin, Ling Li

**Affiliations:** ^1^Key Laboratory for NeuroInformation of Ministry of Education, High-Field Magnetic Resonance Brain Imaging Key Laboratory of Sichuan Province, Center for Information in Medicine, School of Life Science and Technology, University of Electronic Science and Technology of China, Chengdu, China; ^2^School of Foreign Language, Southwest Petroleum University, Chengdu, China

**Keywords:** Broca’s area, speech production, transcranial magnetic stimulation, picture naming, Mandarin Chinese

## Abstract

Although Broca’s area is widely recognized to play an important role in speech production, neuroscientists still debate on its timing recruitment across different languages. In order to investigate the precise time course of phonological encoding for Mandarin Chinese, we applied real triple-pulse transcranial magnetic stimulation (tpTMS) and sham tpTMS within Broca’s area at five different time windows respectively (150 ms, 225 ms, 300 ms, 400 ms and 500 ms) in picture naming task in Mandarin Chinese. To exclude unspecific TMS effects and to make sure that the effects observed in the study were really due to stimulation at Broca’s area, we also conducted a control experiment by a different group of subjects. Significant increases in reaction times (RTs) were observed when real TMS stimulation at Broca’s area was applied at 225 ms, 300 ms and 400 ms time windows with a peak at 225 ms, compared with sham TMS stimulation at other time windows. Our findings support the hypothesis that the phonological encoding in speech production for Chinese language may approximately start from 200 ms and end around 400 ms post target onset, a little earlier than that from 355 ms to 455 ms for Indo-European languages.

## Introduction

Spoken word production involves the coordination of several consecutive processes, such as conceptual preparation, lexical retrieval, phonological encoding, phonetic encoding and articulation. According to an influential language production model (Levelt, 1999), a critical phase in this process is phonological encoding during which the phonological form of the target needs to be prepared to guide the subsequent articulatory movement (*syllabic encoding*). The processes of phonological preparation have been referred to as phonological encoding (Wong et al., 2016). Based on a meta-analysis, Indefrey and Levelt (2004) estimated that the time course of phonological encoding starts from 355 ms and ends around 455–475 ms during the process of word production (Indefrey and Levelt, 2004), and this estimate has been widely supported by many studies of Indo-European languages (Salmelin et al., 1994; Sörös et al., 2003; Vihla et al., 2006; Hultén et al., 2009; Schuhmann et al., 2009).

Nevertheless, it is not self-evident that the above findings from Indo-European languages can be generalized across all world languages. O’Seaghdha et al. (2010) proposed a “proximate unit” principle in process of phonological encoding, according to which languages differ in the phonological unites that are directly connected to lexeme or morpheme nodes in the form network. The proximate units are phonemic segments and metrical frames for Dutch and English (Levelt, 1999), atonal phonological syllables and tonal frames for Mandarin Chinese (Chen et al., 2002). Indeed, studies on Mandarin and Cantonese spoken word production failed to find convergent results with studies on Indo-European languages concerning the time course of phonological encoding in word production (Qu et al., 2012; Yu et al., 2014; Wang et al., 2017). Yu et al. (2014), for instance, investigated the phonological processes in overt Mandarin speech production using a picture naming task together with concurrent recording of event-related potentials (ERPs). More positive ERPs signals evoked by phonologically related words were observed from 180 ms to 300 ms post target presentation, indicating that the time window (180 ms to 300 ms) is the critical period for phonological encoding for Mandarin Chinese. More recently, another study with similar picture naming paradigm was conducted by Wang et al. (2017) with ERP recording. A significant phonological-related ERP effect was found in the time window of 200–400 ms after picture onset. Similarly, in a color adjective-noun picture naming task, Qu et al. (2012) found more positive ERPs in the phonologically related condition in the 200- to 300-ms time window after picture onset. Taken collectively, these findings provide empirical evidences for language-specific aspects in the process of phonological encoding.

Most of recent studies on speech production are conducted by way of magnetoencephalography (MEG) and electroencephalography (EEG) for their high temporal resolution, but these techniques might not be the optimal tools to systematically investigate language function at each particular region or time point. Nevertheless, transcranial magnetic stimulation (TMS) might be a valuable tool to transiently disrupt or even enhance language function during task performance. More importantly, it can be used by changing its stimulation pulse onset and by analyzing stimulation-related impairment of language function at each particular time point and region (Indefrey, 2011; Krieger-Redwood and Jefferies, 2014; Krieg et al., 2016; Sollmann et al., 2017). In this sense, some TMS-related studies have already been conducted to investigate the influence of stimulation onset on picture naming task (Schuhmann et al., 2009, 2012; Wheat et al., 2013; Krieger-Redwood and Jefferies, 2014; Krieg et al., 2016; Sollmann et al., 2017). For example, Schuhmann et al. (2009) applied TMS pulses at five different time points (150 ms, 225 ms, 300 ms, 400 ms and 525 ms) at Broca’s area after picture presentation, and reported an increase in picture naming latency only at 300 ms time window with no such effect in other time points. A follow-up study of Schuhmann et al. (2012) again applied TMS to Broca’s area, but they also applied it to Wernicke’s area, as well as to the midsection of the left middle temporal gyrus (MTG). They found that the midsection of the left MTG becomes functionally relevant at 225 ms after picture onset, followed by Broca’s area at 300 ms and Wernicke’s area at 400 ms. Furthermore, in a study performed by Wheat et al. (2013), TMS pulses were delivered to Broca’s area 75–500 ms after stimulus onset during reading and picture naming and as a result, the reaction times (RTs) were slowest for picture naming when TMS pulses were applied at 300 ms after picture onset. Therefore, TMS is an effective way in investigating the time course of word production.

Broca’s area is widely acknowledged to be related with the core process of phonological encoding (Salmelin et al., 1994; Sörös et al., 2003; Vihla et al., 2006; Hultén et al., 2009). Moreover, a high degree of structural and functional heterogeneity in Broca’s region has been reported cross-linguistically in many studies (Tan et al., 2005; Wu et al., 2012; Zhu et al., 2015). However, most previous studies with TMS technique mainly focused on the alphabetic languages such as English and Dutch (Schuhmann et al., 2009, 2012; Krieger-Redwood and Jefferies, 2014; Krieg et al., 2016; Sollmann et al., 2017), with little effort given to Mandarin Chinese. As mentioned above, phonemic segments and phonological syllables play a different role in the process of phonological encoding between Mandarin Chinese and alphabetic languages like English and Dutch. The aim of the current study was to investigate whether TMS stimulation at Broca’s area for Chinese speakers reveals different timing recruitment from that for alphabetic language speakers such as English and Dutch. To make a comparison between Chinese and alphabetic languages such as Dutch, in the present study, we used Schuhmann et al. (2009) paradigm to apply real and sham TMS stimulation with the same stimulation time windows as that for Dutch (stimulation at 150 ms, 225 ms, 300 ms, 400 ms and 525 ms after stimulus presentation) at Broca’s area, to probe the time course of phonological encoding for Mandarin Chinese. However, except Broca’s area, there was no other active stimulation site in Schuhmann’s study. To exclude unspecific TMS effects, such as the distraction of the TMS coil on the participants head or the presence of the experimenter standing behind the participant and to make sure that the effects observed in the following study were really due to stimulation at Broca’s area, we conducted a control experiment with triple-pulse transcranial magnetic stimulation (tpTMS) stimulation at Vertex and tpTMS sham stimulation at Broca’s area by a different group of subjects. In the light of prior EEG and MEG findings of cross-linguistic difference in the latency of phonological encoding, we propose a hypothesis that the phonological encoding in word production for Chinese language may approximately start from 200 ms and end around 400 ms post target onset, a little earlier than that from 355-ms to 455-ms for Indo-European languages.

## Materials and Methods

### Participants

In the main experiment, participants were 22 healthy volunteers native Chinese speakers, with no history of speaking disorder, 12 of whom were male (Mean age = 23.7 years; Standard Deviation (SD) = 1.35). Eleven subjects were participated in the control experiment (six females, mean age = 22.3 years; SD = 1.22). All participants were right handed, had normal or corrected-to-normal vision and had no history of neurological or psychiatric disorders. Standard exclusion criteria for TMS were applied: pregnancy, metallic implant, cardiac or neurological health condition and specific medication. All participants gave written informed consent before the experiment and received monetary rewards after the experiment. The TMS session was performed according to the published safety guideline. All participants tolerated the TMS procedure well and did not report any adverse effects. The subjects were recruited from the University of Electronic Science and Technology of China. This experiment was carried out in accordance with the recommendations of “the Guideline for Human Behavior Studies, the Institutional Review Board of UESTC MRI Research Center for Brain Research” with written informed consent from all participants. The protocol was approved by the Institutional Review Board of UESTC MRI Research Center for Brain Research.

### Stimulus Materials

Twenty simple white-on-black line drawings corresponding to double-word Chinese nouns as target pictures were selected from a data base of standardized pictures for picture naming tasks in Mandarin production (Qingfang and Yufang, 2003). All target pictures were of moderate word frequency and familiarity as determined by studies of Qingfang and Yufang (2003) on picture naming latency during Chinese language production.

### Experimental Procedures

The main experiment compared the effect of real vs. sham TMS over Broca’s area at different time points of stimulation following picture onset in the process of picture naming in Mandarin language. It entailed a 2 × 5 design with two stimulation type (real TMS vs. sham TMS) and five different time points of stimulation after picture onset as the two within-participant factors. We applied triple-pulse real TMS and triple-pulse sham TMS in two separate sessions on two separate days respectively, during the execution of behaviorally controlled picture naming task in Chinese. The sequence of stimulation type was counterbalanced across participants. The study design enabled us to test for stimulation type and time effects of a controlled neutral activity disruption on Chinese picture naming latencies.

**Figure 1:**
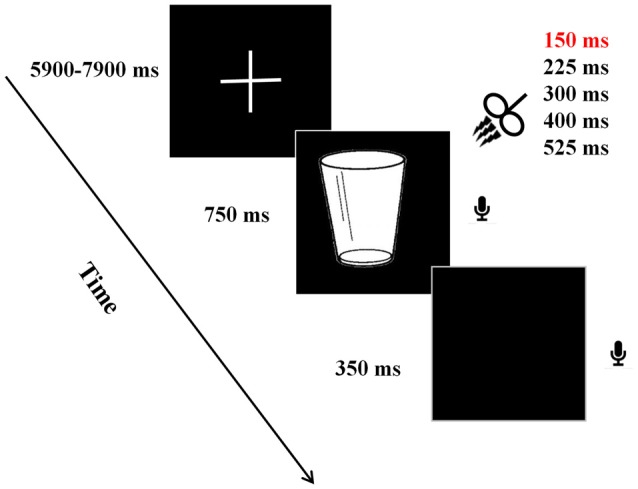
Experimental paradigm. A new trial began with a fixation cross presented between 5900 ms and 7900 ms. Thereafter, a target picture was presented for 750 ms, followed by a blank screen for 350 ms. During the presentation of picture, triple pulse transcranial magnetic stimulation (TMS) stimulation was applied at one of the five time windows in a random way. Participants were instructed to name the presented picture as quickly as possible using a microphone placed in front of each participant. After a jittered delay between 6 s and 8 s, the next trial began.

Presented at a distance 60 cm, the stimuli were displayed in the center of a DELL monitor with the resolution of 1024 × 768 pixels at refresh rate of 60 Hz. A new trial began with a fixation cross presented between 5900 ms and 7900 ms. Thereafter, a target picture was presented for 750 ms, followed by a blank screen for 350 ms. Participants were instructed to name the presented picture as quickly as possible. Verbal responses were recorded by using a microphone placed in front of each participant. After a jittered delay between 6 s and 8 s, the next trial began (see Figure 1).

In order to exclude unspecific TMS effects, a control experiment was performed in another group of subjects. Participants performed the practice and TMS sessions, with real tpTMS stimulation applied to the Vertex and sham tpTMS at Broca’s area.

### TMS Protocol and Stimulation Sites

TMS was applied by using a Magstim super rapid magnetic stimulator and an air-cooled figure-of-eight coil having an outer winding diameter of 70 mm (Magstim Company Limited, Whiteland, UK). A specific figure-of-eight placebo coil (MC-P-B70) was also employed in order to reproduce the same acoustic stimulation as the active coil while not inducing the magnetic field (sham stimulation).

The coils were manually held tangentially to the skull with the coil handle oriented perpendicular to the opercular part of the inferior frontal gyrus using the online visualization function of the BrainSight frameless stereotaxy system (BrainSight Frameless, Rogue Research, Montreal, QC, Canada). tpTMS was applied with an interpulse-interval of 25 ms (40 Hz) at 100% motor threshold (MT). MT was established at the criterion of the lowest intensity of single-pulse TMS stimulation required to evoke motor potentials of at least 50 μv in 5 out of 10 trials, in the contralateral first dorsal interosseous (FDI) muscle following stimulation over the hand area of the participant’s right motor cortex. The average MT values were 52 ± 3.1% of the stimulator’s max output power. The maximum output of the coil and stimulator combination was appropriately 0.8 Tesla and 102 A/μs. The mean stimulation intensity of the tpTMS was about 53.04 A/μs.

Since participants may experience a degree of discomfort or even pain when tpTMS is applied over Broca’s area, we delivered a test pulse prior to the experimental session and asked the participants whether they felt the pulse aversive. All the participants tolerated the tpTMS well and did not ask to stop the experiment nor did they pull their head away from the coil during the stimulation.

**Figure 2:**
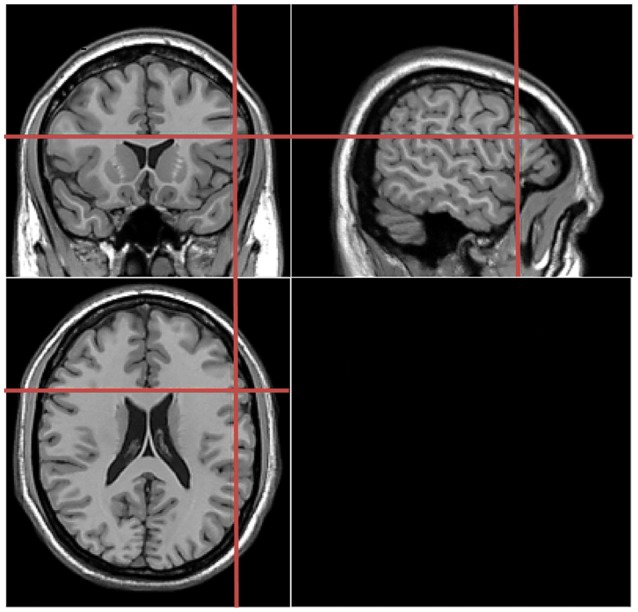
Coronal, axial, sagittal views of the stimulated site left inferior frontal gyrus, Montreal Neurological Institute (MNI) coordinates: −55, 18, 21, depicted on a standard template from MRIcro.

Stimulation locations were targeted via the BrainSight stereotaxic neuronavigator (Rogue Research, Montreal, QC, Canada), equipped with a Polaris Vicra position sensor system. Landmarks on all participants’ heads were co-registered to a standard MRI template using Montreal Neurological Institute (MNI) coordinates. The stimulation sites were determined on the basis of study by Schuhmann et al. (2009), which used behavioral tasks similar to those used in our study. The location of the target stimulation site was centered on the following MNI coordinates: −55, 18, 21 (see Figure 2). In the control experiment, the coordinates for the sham stimulation at Broca’s area was the same to that in the main experiment. For the TMS stimulation over the Vertex (control experiment), this was localized as a midpoint between the inion and the nasion and equidistant from the left and right ear. The TMS coil was placed on the corresponding locations over the participants’ scalp. Brainsight was used to track the position of the TMS coil throughout the stimulation period, ensuring that it remained on the target location.

### TMS Procedure

There were two sessions with event-related real tpTMS stimulation and sham tpTMS stimulation respectively. The time interval between the two TMS sessions was 1 week to make sure that there were no carryover effects between the two sessions. The sequence of stimulation type was counterbalanced across participants. Before starting the main experiment, participants were instructed to practice a training session to be familiar with the stimuli and to name the stimuli repeatedly as to reach a stable performance level in naming latency. Each experimental session consisted of 120 trials, divided into four blocks of 30 trials each.

Event-related tpTMS was applied at five different points in time following picture presentation onset, namely at: (1) 150–175–200 ms; (2) 225–250–275 ms; (3) 300–325–350 ms; (4) 400–425–450 ms; and (5) 525–550–575 ms. The presentation of the pictures and TMS time window conditions were applied randomly across trials within each session.

There were mainly two reasons for the spacing of time windows. First, according to Levelt’s language production model (Levelt, 1999; Indefrey, 2011), estimated onset times for operations in spoken word production are as follows: conceptual preparation takes place within a time frame of 0–200 ms post picture onset. Lemma selection should begin between 150 ms and 200 ms post picture onset and be over at some moment between 150 ms and 350 ms post picture onset. Then phonological code retrieval may begin between 275 ms and 355 ms after picture onset. Syllabification takes place from 355 ms to 455 ms post picture onset. Finally, phonetic encoding may begin between 455 ms and 600 ms followed by articulation at about 600 ms. In order to investigate the timing recruitment of Broca’s area during the process of spoken word production, we planned to stimulate TMS within different time windows involving the whole process.

Second, several previous studies have applied tpTMS at the five time windows to probe the temporal features of Broca’s area for alphabetic language Dutch (Schuhmann et al., 2009, 2012). The aim of current study was to investigate whether there are different timing recruitments of Bora’s area for Mandarin Chinese, compared with that of alphabetic languages such as Dutch and English. To make a good comparison between Mandarin Chinese and Dutch, we kept the same stimulation time window with that of Schuhmann’s study.

### Response Time Measures

**Figure 3:**
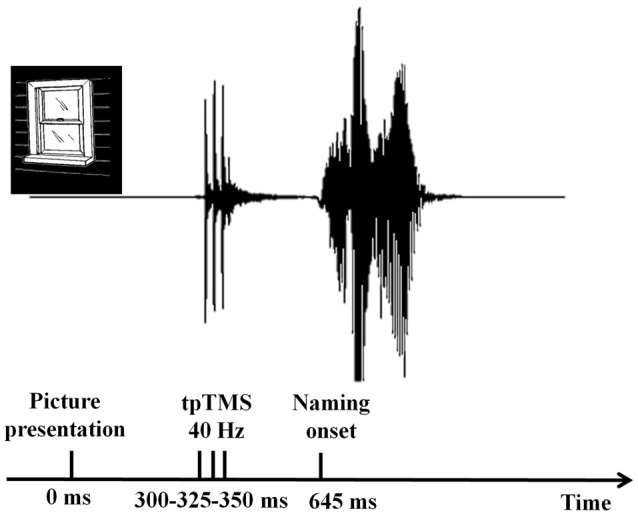
Verbal response time analyses. The latency of the verbal responses was measured using the speech wave envelopes with digital audio editing software cooledit v2.1. Naming onset was defined as the first detectable amplitude in the speech wave envelop.

Verbal responses were recorded by using a microphone placed in front of each participant. The latency of the verbal responses was measured using the speech wave envelopes with digital audio editing software cooledit v2.1. Acoustic information was digitized at an 8-bit resolution with a sampling rate of 22 kHz. An amplitude filter was used to reduce the noise before the determination of the speech onset (see Figure 3).

The accuracy of verbal responses was checked offline by listening to audio recordings. One participant made a large number of errors due to discomfort of strong contractions of face muscles and therefore was excluded from the analysis. Semantically incorrect responses, as well as responses of hesitation, or the “tip of the tongue phenomenon” and extreme delay which exceeded more than two SDs were all excluded from further statistical analysis. This resulted in the removal of ≈4.3% of all responses across all trails.

### Statistical Analysis

RTs and correct response rates (accuracy) were measured. A repeated measures analysis of variance (ANOVA) was performed to compare RTs and accuracies, with stimulation type (real TMS vs. sham TMS) and time window (stimulation at 150 ms, 225 ms, 300 ms, 400 ms and 525 ms after stimulus presentation) as within-subject factors. If necessary, sham TMS and real TMS session were further compared using *post hoc* *t*-tests, Bonferroni corrected for multiple comparisons. The threshold of the *p*-value for the 45 possible comparisons across the 10 conditions (10*9/2 = 45) was adjusted to 0.0011 (0.05/45 = 0.0011).

## Results

Table 1 displays the mean values of the error rates (ERs) and RTs for real and sham TMS stimulation condition.

**Table 1:** Mean values (and SEM) of error rates (ERs) and reaction times (RTs).

	Error rates (%)		RTs (ms)	
	Mean (SEM)	Mean (SEM)	Mean (SEM)	Mean (SEM)
	real	sham	real	sham
150 ms	0.12 (0.02)	0.08 (0.02)	569.48 (9.3)	550.27 (11.44)
225 ms	0.13 (0.02)	0.12 (0.02)	590.06 (9.6)	550.82 (10.15)
300 ms	0.10 (0.02)	0.05 (0.02)	580.36 (9.1)	548.48 (10.71)
400 ms	0.14 (0.03)	0.11 (0.02)	585.61 (7.16)	561.27 (9.61)
525 ms	0.09 (0.02)	0.08 (0.01)	597.94 (8.23)	576.43 (10.51)

### TMS Induced Changes in Accuracy

The mean ERs were calculated for each time window for both the sham and real stimulation. The mean amount of errors during the real TMS experiment ranged from 0.06 (SD = 0.08) up to 0.11 (SD = 0.09) in the different time windows. During the sham TMS experiment, the mean amount of errors was comparable, ranging from 0.10 (SD = 0.09) to 0.15 (SD = 0.12).

A two-factorial ANOVA with stimulation type (real TMS vs. sham TMS) and time window (stimulation at 150 ms, 225 ms, 300 ms, 400 ms and 525 ms after stimulus presentation) as the two within-subject factors did not reveal a significant effect of stimulation type (*F*_(1,21)_ = 2.834 *p* = 0.107), but there was significant effect of time window (*F*_(4,84)_ = 5.227 *p* < 0.05). Moreover, there was no interaction between stimulation type and time window (*F*_(4,84)_ = 0.909 *p* = 0.462).

### TMS Induced Changes in Latency

**Figure 4:**
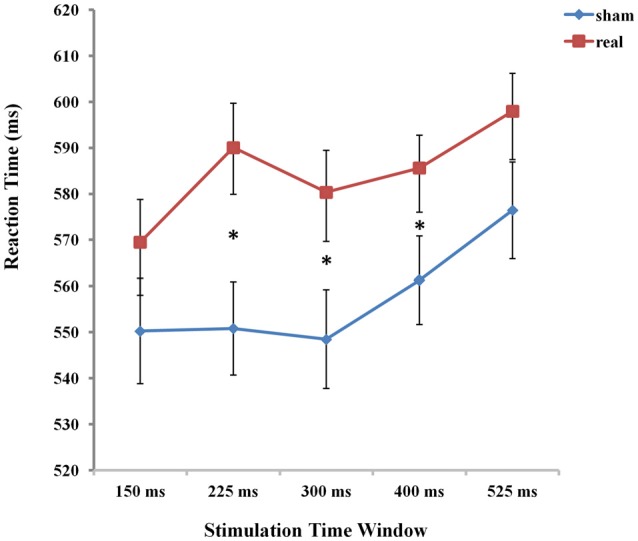
The mean reaction times (RTs) of the five time windows in real and sham stimulation conditions. An asterisk means *p* < 0.05 (Bonferroni corrected).

The two factorial ANOVA with stimulation type (real TMS vs. sham TMS) and time window (stimulation at 150 ms, 225 ms, 300 ms, 400 ms, and 525 ms after stimulus presentation) as the two within-subject factors revealed a main effect of time window (*F*_(4,84)_ = 11.728, *p* < 0.001), indicating that the effect of TMS stimulation over Broca’s area differed between the various time points of stimulation. The analysis also yielded a main effect of stimulation type (*F*_(1,21)_ = 19.002, *p* < 0.001). More importantly, there was an interaction between stimulation type and time windows (*F*_(4,84)_ = 3.660, *p* < 0.05; see Figure 4).

To determine the source of this 2-way interaction, paired *t*-test, Bonferroni corrected for multiple comparisons, were conducted to compare the respective five time points of stimulation between real and sham conditions. The threshold of the *p*-value for the 45 possible comparisons across the 10 conditions (10*9/2 = 45) was adjusted to 0.0011 (0.05/45 = 0.0011). The results showed that RTs were significantly prolonged in the time window of 225 ms, 300 ms and 400 ms compared to the other two time windows of 150 ms and 525 ms between real and sham condition (real: RT = 590.06 ms, sham: RT = 550.82 ms, *t*_(21)_ = −4.553, *p* = 0.0001 for 225 ms; real: RT = 580.36 ms, sham: RT = 548.48 ms, *t*_(21)_ = −3.961, *p* = 0.0001 for 300 ms; real: RT = 585.61 ms, sham: RT = 561.27 ms, *t*_(21)_ = −3.875, *p* = 0.0001 for 400 ms).

To assess the difference between real and sham stimulation at five time windows, we calculated the TMS cost across time window. We compared TMS cost (Broca-sham) using one-way ANOVA with five time windows (150 ms, 225 ms, 300 ms, 400 ms, 525 ms respectively) as factors. There was a significant main effect of time window (*F*_(1,21)_ = 3.660, *p* = 0.008), which shows that real TMS stimulation at Broca’s area resulted in a radical increase in RT at 225 ms after picture onset.

**Figure 5:**
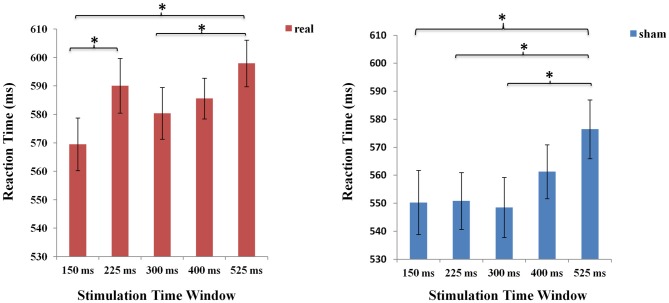
The mean RTs of the five time windows in real and sham stimulation conditions respectively. An asterisk means *p* < 0.05 (Bonferroni corrected).

Meanwhile, separate one-factorial analyses of real and sham TMS were conducted respectively to compare the five different time windows under each stimulation type condition. Under the real stimulation type condition, there was a main effect of time window (*F*_(4,84)_ = 7.920 *p* < 0.001). And results showed that RT rapidly increased at 225 ms compared to the first time window at 150 ms. Moreover, there was an increase in RT at 525 ms compared to time windows at 150 ms (*p* < 0.001). Similarly, under the sham stimulation type condition, results also revealed a significant increase in RT at 525 ms (*p* < 0.05; see Figure 5).

### TMS Effects Between Broca’s Area and Vertex

To test for the differences between TMS stimulation at Broca’s area and vertex, we first calculated the difference Broca’s-sham and vertex-sham for the five time windows. Then, repeated ANOVA was conducted with TMS cost at five time windows (Broca’s-sham and vertex-sham) as within-subject factor and groups (Broca’s group vs. Vertex group) as between-subject factor. Results showed that there was no main effect of TMS cost at five time windows (*F*_(4,124)_ = 0.716 *p* > 0.05), and the between-groups effect was not significant either (*F*_(1,31)_ = 1.840, *p* = 0.185). However, interactions between TMS cost and groups were found (*F*_(4,124)_ = 3.161 *p* = 0.016).

**Figure 6:**
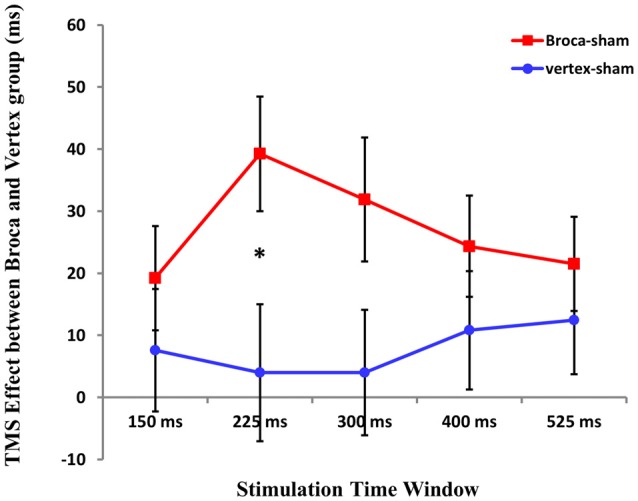
The cost of the TMS effect between Boca’s and Vertex group at five time window. An asterisk means *p* < 0.05 (Bonferroni corrected).

A subsequent two-sample *T* test was applied to compare the TMS cost at five time windows between two groups. Statistical analysis revealed that TMS cost between two groups was only significant at 225 ms time window after picture onset (*t*_(1,31)_ = 2.207 *p* = 0.035; see Figure 6).

## Discussion

In order to investigate the precise time course of phonological encoding for Mandarin Chinese, we applied real TMS and sham TMS within Broca’s area at five different time windows after picture onset. To exclude unspecific TMS effects and to make sure that the effects observed in the study were really due to stimulation at Broca’s area, we also conducted a control experiment with TMS stimulation at Vertex as control site and sham stimulation at Broca’s area by different group of subjects.

First, in the main experiment, the two factorial ANOVA analyses yielded a main effect of stimulation type and interaction between sham and active stimulation at Broca’s area, indicating that stimulation between sham and Broca’s area may have different effect on speech production. Then, in the control experiment, to exclude unspecific factors of TMS stimulation and make sure that the effects observed in the main experiment was really due to Broca’s area, repeated ANOVA of TMS cost in five different time windows was conducted between Broca’s and vertex group. No between-groups effect was found. More importantly, interactions between TMS cost (Broca’s-sham and vertex-sham) and between-groups effect was found, indicating that Broca’s group and Vertex group differed in the time window of interest, especially at the 225 ms time window. Taken the results of main and control experiment together, we drew the conclusion that TMS stimulation at Vertex didn’t cause the same effect as that at Broca’s area. Our data thus confirmed that TMS method in our study did effectively transiently disrupt the process of the language production at Broca’s area. In other words, the TMS-related effects observed in present study really resulted from stimulation at Broca’s area, that is, applying TMS over Broca’s area has an effect on the procedure of naming pictures.

Second, with regard to the timing recruitment of Broca’s area in speech production, significant increases in RTs were observed when real TMS stimulation was applied at 225 ms, 300 ms and 400 ms respectively after picture onset, compared with sham TMS stimulation at other time windows. As mentioned in Introduction, our results are consistent with recent ERP studies on Mandarin and Cantonese Chinese (Qu et al., 2012; Yu et al., 2014; Wang et al., 2017), indicating that the phonological encoding process for Mandarin Chinese at Broca’s area may start between 200 ms and 400 ms.

One point to note is that effects of TMS stimulation at Broca’ area emerging at such time window between 200 ms and 400 ms diverges from the temporal estimate for phonological encoding (355–455 ms) in results of comprehensive meta-analysis for alphabetic languages (Indefrey and Levelt, 2004; Indefrey, 2011). More recently, neuroimaging studies on alphabetic languages have provided supporting evidence that activation in Broca’s area in process of phonological encoding starts after 400 ms, with no activation before 200 ms (Salmelin et al., 1994; Vihla et al., 2006; Hultén et al., 2009; Indefrey, 2011). In another related study applying TMS stimulation at Broca’s area at five time windows, Schuhmann et al. (2009) reported increased naming latencies between 300 ms and 350 ms after picture onset but not before or after. Due to short naming latency of 470 ms, they explained that the effect on phonological encoding within time window of 300–350 ms is consistent with Indefrey’s model for phonological encoding (355–455 ms). Interestingly, using the same experimental paradigm, results of the present study is not compatible with Schuhmann’s study on Dutch but in line with previous studies on Mandarin Chinese with time window for phonological encoding as early as 180–300 ms (Yu et al., 2014), 200–300 ms (Qu et al., 2012) and 200–400 ms (Wang et al., 2017).

One explanation for the discrepancy between alphabetic and non-alphabetic languages invokes the cross-linguistic differences on the process of phonological encoding. For instance, as a non-alphabetic language, Chinese maps each graphical character directly into one syllable using orthography-to-phonology transformation. Whereas, alphabetic languages like German and English segment each word into letters and then translate into a phonetic sequence following the grapheme-to-phoneme conversion rules (Tan et al., 2005). According to the WEAVER++model by Roelofs (1992, 1997), the form network in word production for typical alphabetic languages encompasses three levels: (1) the activated morpheme; (2) segments corresponding to the target name; simultaneously, a metrical frame which conveys information about stress patterns; and (3) segments and metrical frame are merged into “syllable motor programs”. This model has been largely supported by a large amount of studies on alphabetic language such as English and Dutch (Meyer, 1990; Schriefers et al., 1990). Nevertheless, studies on Mandarin and Cantonese spoken word production failed to find supporting evidence at segment level (Chen et al., 2002; O’Seaghdha et al., 2010; You et al., 2012). To account for the cross-linguistic differences in phonological encoding, O’Seaghdha et al. (2010) proposed a “proximate unit” principle according to which phonemes are the proximate units for Indo-European languages, whereas syllables are for Mandarin Chinese. By the same token, more recently, Roelofs (2015) postulated the form network for Mandarin Chinese the following four levels: (1) the activated morpheme; (2) atonal syllable nodes are activated, simultaneously, a tonal frame is activated; (3) segments corresponding to the target name; and (4) segments and metrical frame are merged into “syllable motor programs”. If this is the case, then the phonological encoding for Mandarin Chinese would involve an extra step compared with that for Indo-European languages (Roelofs, 2015). Therefore, with similar naming latencies of 600 ms in picture naming task between alphabetic and non-alphabetic languages, it is reasonable that the time course of phonological encoding for Mandarin Chinese is between 200 ms and 400 ms, a little earlier than that for alphabetic languages.

In addition, we also found that both sham and real stimulation over Broca’s area resulted in an increase in RTs at 525 ms after picture presentation. However, there is no significant effect of TMS stimulation between sham and real stimulation at 525 ms after picture presentation. It may be due to the fact that the mean RTs for speech production is around 556 ms, so delivering tpTMS at 525 ms may inevitably interfere with the process of speech production, which may result in an increase in RTs for real and sham stimulation respectively. Thus, future study should exclude those disturbances from investigation.

One limitation of the current study is that we used different group of subjects in the control experiment with smaller sample size of subjects. And this may affect the validity of the study. Future studies should design within-subject paradigm with large sample of subjects to investigate the process of speech production for Chinese.

In summary, our findings are in line with previous studies in Mandarin Chinese, confirming the hypothesis that the phonological encoding in word production for Chinese language may approximately start from 200 ms and end around 400 ms post target onset, a little earlier than that from 355 ms to 455 ms for Indo-European languages.

## Author Contributions

QZ performed research, analyzed data and wrote the main manuscript text. BY revised the manuscript. JZ performed research. ZJ prepared Figures 1–6. LL designed research. All authors reviewed the manuscript.

## Conflict of Interest Statement

The authors declare that the research was conducted in the absence of any commercial or financial relationships that could be construed as a potential conflict of interest.
